# Accuracy of 11 Wearable, Nearable, and Airable Consumer Sleep Trackers: Prospective Multicenter Validation Study

**DOI:** 10.2196/50983

**Published:** 2023-11-02

**Authors:** Taeyoung Lee, Younghoon Cho, Kwang Su Cha, Jinhwan Jung, Jungim Cho, Hyunggug Kim, Daewoo Kim, Joonki Hong, Dongheon Lee, Moonsik Keum, Clete A Kushida, In-Young Yoon, Jeong-Whun Kim

**Affiliations:** 1 Asleep Co., Ltd. Seoul Republic of Korea; 2 Clionic Lifecare Clinic Seoul Republic of Korea; 3 Department of Psychiatry and Behavioral Sciences Stanford University Medical Center Redwood City, CA United States; 4 Department of Psychiatry Seoul National University Bundang Hospital Seongnam-si Republic of Korea; 5 Department of Otorhinolaryngology-Head and Neck Surgery Seoul National University Bundang Hospital Seongnam-si Republic of Korea

**Keywords:** consumer sleep trackers, wearables, nearables, airables, sleep monitoring, sleep stage, comparative study, polysomnography, multicenter study, deep learning, artificial intelligence, Fitbit Sense 2, Amazon Halo Rise, SleepRoutine

## Abstract

**Background:**

Consumer sleep trackers (CSTs) have gained significant popularity because they enable individuals to conveniently monitor and analyze their sleep. However, limited studies have comprehensively validated the performance of widely used CSTs. Our study therefore investigated popular CSTs based on various biosignals and algorithms by assessing the agreement with polysomnography.

**Objective:**

This study aimed to validate the accuracy of various types of CSTs through a comparison with in-lab polysomnography. Additionally, by including widely used CSTs and conducting a multicenter study with a large sample size, this study seeks to provide comprehensive insights into the performance and applicability of these CSTs for sleep monitoring in a hospital environment.

**Methods:**

The study analyzed 11 commercially available CSTs, including 5 wearables (Google Pixel Watch, Galaxy Watch 5, Fitbit Sense 2, Apple Watch 8, and Oura Ring 3), 3 nearables (Withings Sleep Tracking Mat, Google Nest Hub 2, and Amazon Halo Rise), and 3 airables (SleepRoutine, SleepScore, and Pillow). The 11 CSTs were divided into 2 groups, ensuring maximum inclusion while avoiding interference between the CSTs within each group. Each group (comprising 8 CSTs) was also compared via polysomnography.

**Results:**

The study enrolled 75 participants from a tertiary hospital and a primary sleep-specialized clinic in Korea. Across the 2 centers, we collected a total of 3890 hours of sleep sessions based on 11 CSTs, along with 543 hours of polysomnography recordings. Each CST sleep recording covered an average of 353 hours. We analyzed a total of 349,114 epochs from the 11 CSTs compared with polysomnography, where epoch-by-epoch agreement in sleep stage classification showed substantial performance variation. More specifically, the highest macro F1 score was 0.69, while the lowest macro F1 score was 0.26. Various sleep trackers exhibited diverse performances across sleep stages, with SleepRoutine excelling in the wake and rapid eye movement stages, and wearables like Google Pixel Watch and Fitbit Sense 2 showing superiority in the deep stage. There was a distinct trend in sleep measure estimation according to the type of device. Wearables showed high proportional bias in sleep efficiency, while nearables exhibited high proportional bias in sleep latency. Subgroup analyses of sleep trackers revealed variations in macro F1 scores based on factors, such as BMI, sleep efficiency, and apnea-hypopnea index, while the differences between male and female subgroups were minimal.

**Conclusions:**

Our study showed that among the 11 CSTs examined, specific CSTs showed substantial agreement with polysomnography, indicating their potential application in sleep monitoring, while other CSTs were partially consistent with polysomnography. This study offers insights into the strengths of CSTs within the 3 different classes for individuals interested in wellness who wish to understand and proactively manage their own sleep.

## Introduction

With the growing recognition of the importance of sleep for overall health [1], there has been a significant rise in public interest in monitoring sleep patterns using consumer sleep trackers (CSTs) [2-4]. While the laboratory monitoring of sleep using the traditional sleep analysis tool polysomnography has several limitations associated with the need for cumbersome sensors [5], CSTs facilitate individual monitoring of sleep at home using minimal equipment. Recently, many big tech companies, including Apple, Samsung, and Google, as well as health care startups like Withings and Oura, have released their own CSTs. These companies have made significant contributions to enhancing the performance of CSTs by integrating deep learning algorithms and biosignal sensing technologies [6,7]. As a result, CSTs have emerged as accessible solutions for home sleep monitoring [2,6,8-11]. CSTs are widely used by not only individuals interested in wellness who wish to understand and proactively manage their own sleep, but also those who want to self-check and screen for sleep disorders.

This study classified CSTs into 3 types: wearables, nearables, and airables. Wearable devices or wearables, such as smartwatches and ring-shaped devices, are generally worn by users to track sleep using sensors like photoplethysmography sensors and accelerometers [6,12-16]. Nearable devices or nearables, placed near the body without direct contact, have radar or mattress pads to detect subtle movements during sleep [9,17]. Airable devices or airables use mobile phones to analyze sleep via built-in microphones or environmental sensors [2,3,8]. This classification is based on the measurement methods and biological signals used in each category.

Given the surge of diverse CSTs, it is necessary to conduct comprehensive and objective evaluations of the performance of these CSTs available in the market [4,15,18-21]. Some studies compared CSTs and alternative tools available for sleep analysis, such as electroencephalography headbands [4] or subjective sleep diaries [12] (without employing the gold standard polysomnography), which failed to validate the consistency between CSTs and polysomnography. Chinoy et al [11] compared the performance of 7 CSTs with polysomnography (Fatigue Science Readiband, Fitbit Alta HR, Garmin Fenix 5S, Garmin Vivosmart 3, EarlySense Live, ResMed S+, and SleepScore Max). However, this previous study showed limitations of recruitment from a single institution and exclusion of widely used CSTs available commercially.

To address these limitations, we conducted a multicenter study comparing widely used or newly released CSTs with in-lab polysomnography in a hospital setting. By simultaneously assessing multiple CSTs, we aimed to minimize bias and evaluate their performance across various metrics. Subgroup analysis was also performed to assess the impact of demographic factors on performance, including sex assigned at birth, apnea-hypopnea index (AHI), and BMI. By performing the most extensive simultaneous comparison of widely used CSTs and conducting a multicenter study with diverse demographic groups, this study offers comprehensive insights into the performance and applicability of these CSTs for sleep monitoring.

## Methods

### Participants

The demographic information of the study participants is presented in Table 1. A total of 75 individuals were recruited from Seoul National University Bundang Hospital (SNUBH) and Clionic Lifecare Clinic (CLC). Of these 75 individuals, 37 (27 males and 10 females) with scheduled polysomnography for sleep disorders were recruited from SNUBH and 38 (12 males and 26 females) were recruited through an online platform from CLC. Both institutions used the same inclusion criteria, including age between 19 and 70 years and presence of subjective sleep discomfort. Individuals with uncontrolled acute respiratory conditions were excluded. Participant demographics revealed that the sample consisted of 52% (39/75) males, with a mean age of 43.59 (SD 14.10) years and a mean BMI of 23.90 (SD 4.07) kg/m^2^. Significant differences in sleep measures were observed between the 2 institutions, including time in bed, total sleep time, wake after sleep onset (WASO), and AHI. Multimedia Appendix 1 presents the number of measurements and data collection success rate for each CST.

**Table 1:** Comparative analysis of participant demographics.

Characteristic	Total (N=75)	SNUBH^a^ (n=37)	CLC^b^ (n=38)	*P* value^c^
Male, n (%)	39 (52)	27 (73)	12 (32)	<.001^d^
Age (years), mean (SD)	43.59 (14.10)	53.49 (11.96)	33.95 (8.07)	<.001^d^
BMI (kg/m^2^), mean (SD)	23.90 (4.07)	24.64 (4.07)	23.18 (3.98)	.12
Time in bed (hours), mean (SD)	7.24 (0.92)	8.01 (0.31)	6.49 (0.66)	<.001^d^
Total sleep time (hours), mean (SD)	5.82 (1.33)	6.26 (1.2)	5.40 (1.33)	.005^d^
Sleep latency (hours), mean (SD)	0.27 (0.37)	0.27 (0.38)	0.26 (0.38)	.93
Wake after sleep onset (hours), mean (SD)	1.15 (1.2)	1.48 (1.22)	0.83 (1.23)	.02^d^
Sleep efficiency (%), mean (SD)	81.00 (17.5)	78.40 (15.54)	83.54 (19.1)	.20
Apnea-hypopnea index, mean (SD)	18.18 (20.39)	26.56 (24.25)	10.02 (10.99)	<.001^d^

^a^SNUBH: Seoul National University Bundang Hospital.

^b^CLC: Clionic Lifecare Clinic.

^c^All *P* values were obtained using 2-sample independent *t* tests. For the male category, Fisher exact test was applied.

^d^Statistical significance (*P*<.05).

### Evaluation of CSTs

We evaluated 11 different CSTs in this study. Wearables included ring-type devices (Oura Ring 3, Oura) and watch-type devices (Apple Watch 8, Apple Inc; Galaxy Watch 5, Samsung Electronics Co, Ltd; Fitbit Sense 2, Fitbit Inc; and Google Pixel Watch, Google LLC). Nearables included pad-type devices (Withings Sleep Tracking Mat, Withings) and motion sensor devices (Amazon Halo Rise, Amazon Inc; and Google Nest Hub 2, Google LLC). Airables included mobile apps (SleepRoutine, Asleep; SleepScore App, SleepScore Labs; and Pillow, Neybox Digital Ltd) with iPhone 12s and Galaxy S21s. The selection of these devices was based on their popularity and availability in the market at the time of the study. The methods of usage and application for each sleep tracker were based on user instructions provided by the respective manufacturers. To mitigate a possible learning curve for each device, the researchers educated participants on how to use each device before measurements, and in the case of wearable CSTs, they ensured that the devices were properly fitted. During the study, software updates of all devices were performed on March 1, 2023, to ensure that they were up-to-date, and automatic updates were disabled.

### Study Design

This was a prospective cross-sectional study conducted to investigate the accuracy of various CSTs and polysomnography in analyzing sleep stages. It was conducted at 2 independent medical institutions in South Korea, namely SNUBH, a tertiary care hospital, and CLC, a primary care clinic.

All participants were contacted by phone at least 2 days prior to participating in the polysomnography study and were provided with instructions. On the day before and the day of the test, they were advised to abstain from alcohol and caffeine consumption and refrain from engaging in strenuous exercise, and were informed of the designated test time. These measures were taken to standardize participant behaviors and minimize the influence of potential confounding factors. On the designated test days, participants visited the hospitals and received detailed explanations about the study. They provided written informed consent and underwent polysomnography at each institution. Polysomnography recordings were conducted in a controlled sleep laboratory environment in accordance with the guidelines recommended by the American Academy of Sleep Medicine (AASM) [22]. Two technicians independently interpreted the results, followed by a review by sleep physicians.

**Figure 1:**
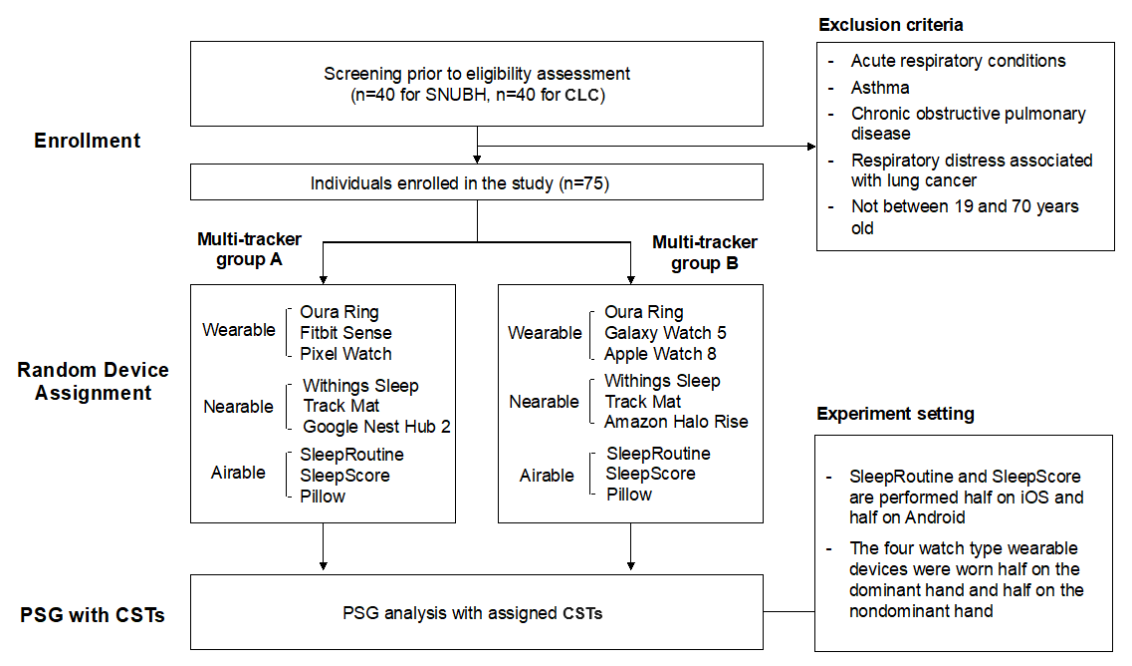
Flowchart outlining the experimental design. Experimental procedures involving subject enrollment, CST assignment, and experimental settings for simultaneous measurement involving both CSTs and PSG. CLC: Clionic Lifecare Clinic; CST: consumer sleep tracker; PSG: polysomnography; SNUBH: Seoul National University Bundang Hospital.

**Figure 2:**
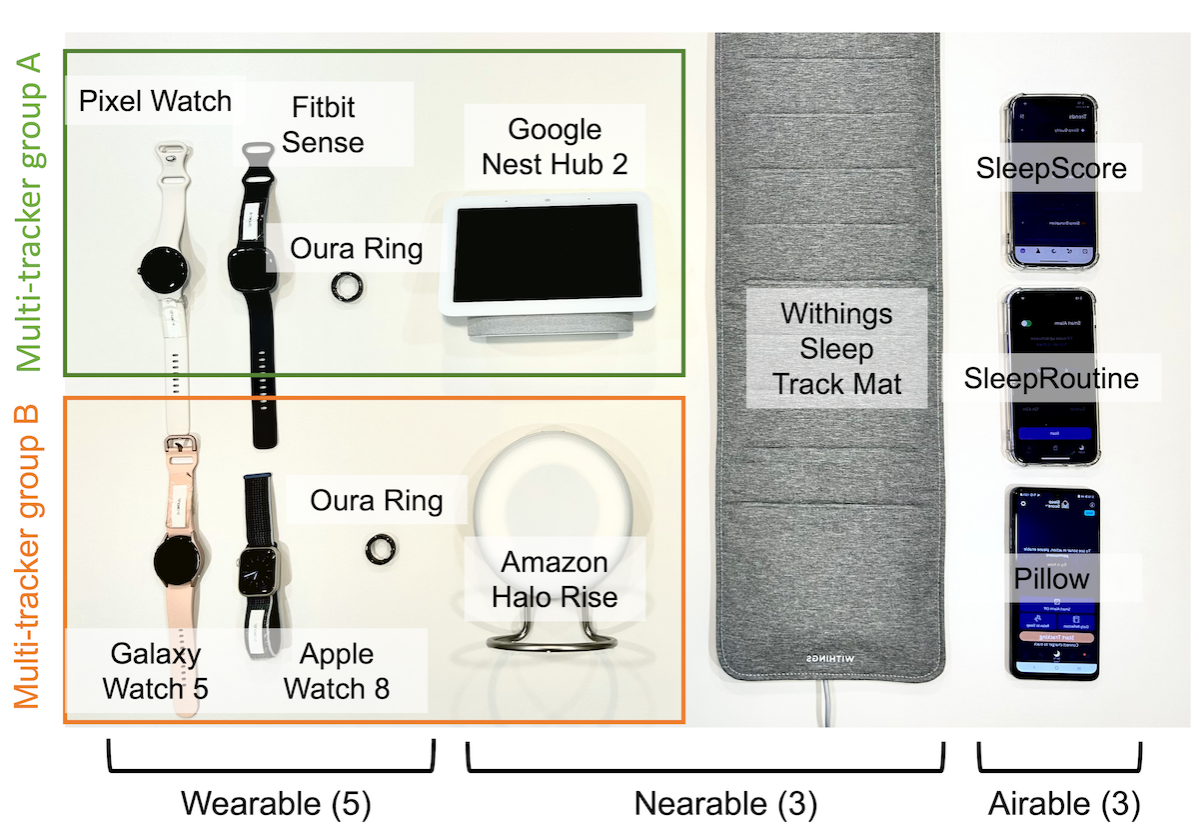
Configuration of consumer sleep trackers used in the experiment.

To address the issue of interference due to multiple CSTs sharing the same biosignals, the participants were divided into 2 groups in both medical institutions: multi-tracker group A and multi-tracker group B, as illustrated in Figure 1. The configurations of the CSTs are presented in Figure 2. Specifically, at SNUBH, multi-tracker group A consisted of 18 individuals and multi-tracker group B consisted of 19 individuals. Similarly, at CLC, each group included 19 individuals. Across both institutions, the demographic statistics for participants in multi-tracker groups A and B demonstrated no significant differences across all metrics, as presented in Multimedia Appendix 2. Each group included a combination of noninterfering CSTs. Specifically, the nearables Google Nest Hub 2 and Amazon Halo Rise, which use similar radar sensors to detect motion, were allocated to different groups. In the case of wearables, participants were allowed to simultaneously wear a maximum of 2 watch devices, which are a type of wearable, with 1 on each wrist. Consequently, Fitbit Sense 2 and Pixel Watch were assigned to multi-tracker group A, while Galaxy Watch 5 and Apple Watch 8 were assigned to multi-tracker group B. As a result, these devices were expected to yield approximately half of the intended measurements. Airables, which were available on both iOS and Android devices (SleepRoutine and SleepScore), were analyzed, with half of them on iOS and the other half on Android. We used Pillow on iOS, as it is not available on Android. The polysomnography and CST results were then compared and analyzed.

### Ethics Approval

Ethics approval was obtained from the respective Institutional Review Board (IRB) of each institution (IRB number B-2302-908-301 from SNUBH and number P01-202302-01-048 from CLC).

### Statistical Methods and Evaluation Metrics

Two-sample independent *t* tests were employed to compare demographic information (Table 1) and sleep measures, and significance was determined based on a *P* value of <.05. The average sleep measurements were compared, and any proportional bias was assessed using Pearson correlation and a Bland-Altman plot. The study used sensitivity, specificity, and F1 scores as evaluation metrics for sleep stage classification. Macro F1 scores, weighted F1 scores, and kappa values were used to summarize the results of the evaluation, considering the imbalance in data classes, such as sleep stages. All statistical analyses and visualizations were conducted using Python 3 (version 3.9.16) and used the scikit-learn, matplotlib, and scipy libraries.

### Data Preprocessing

Three main steps were followed in the data processing stage. First, raw sleep score data were extracted from each CST device, either through direct download via the manufacturer’s app or the web portal, or by requesting raw data from the SleepRoutine device manufacturer. The sleep score codes were standardized across devices, with the wake stage assigned 0, the light stage assigned 1, the deep stage assigned 2, and the rapid eye movement (REM) stage assigned 3. Apple Watch 8 used alternative expressions, such as “core sleep” instead of light sleep.

The extracted data were synchronized in time to compare CST results of sleep tracking with polysomnography accurately. The sleep stages measured by devices earlier than polysomnography scores were discarded. Conversely, for devices that started measuring after polysomnography scoring, the sleep stages were marked as the wake stage until the measurement began. The end point of all device measurements was aligned with the end of polysomnography, resulting in consistent measurement of total time in bed across the devices. 

Because the sleep stages changed at every epoch, the results may be inaccurate if the start time of the 30-second epoch differed. Therefore, the results of sleep stages involving all devices, including polysomnography, were segmented into 1-second intervals and compared every second. This approach enabled a more precise comparison of sleep stage results between polysomnography and CST measurements, and eliminated potential bias.

## Results

### Epoch-by-Epoch Analysis: Overall Performance

Table 2 presents the results of epoch-by-epoch agreements between polysomnography and each of the 11 CSTs under the sleep stage classification. SleepRoutine (airable) demonstrated the highest macro F1 score of 0.6863, which was closely followed by Amazon Halo Rise (nearable), with a macro F1 score of 0.6242. In terms of Cohen κ, a measure of interrater agreement, 3 wearables (Google Pixel Watch, Galaxy Watch 5, and Fitbit Sense 2), 1 nearable (Amazon Halo Rise), and 1 airable (SleepRoutine) demonstrated moderate agreement with sleep stage classification (κ=0.4-0.6). On the other hand, 2 wearables (Apple Watch 8 and Oura Ring 3), 1 nearable (Withings Sleep Tracking Mat), and 1 airable (SleepScore) showed a fair level of agreement (κ=0.2-0.4). Finally, Google Nest Hub 2 (nearable) and Pillow (airable) exhibited only a slight level of agreement across sleep stage classifications. The performance of CSTs was assessed in 2 distinct institutions, where the macro F1 scores, averaged over all devices in each institution, were 0.4973 and 0.4876 at SNUBH and CLC, respectively. There was no significant difference in performance between these 2 locations. Among the 11 CSTs evaluated, 5 (Galaxy Watch 5, Apple Watch 8, Amazon Halo Rise, Pillow, and SleepRoutine) exhibited better performance at SNUBH, while the remaining CSTs demonstrated superior performance at CLC.

**Table 2:** Epoch-by-epoch agreement: classification of 4 sleep stages.

Variable		Overall					SNUBH^a^		CLC^b^	
		Accuracy	Weighted F1	Cohen κ	Macro F1	Macro F1		Macro F1		
**Airable**										
	SleepRoutine (n=67)^c^	0.7106^d^	0.7166^d^	0.5565^d^	0.6863^d^	0.7188^d^		0.6551^d^		
	SleepScore (n=38)	0.4329	0.4472	0.2065	0.4049	0.4408		0.3094		
	Pillow (n=74)	0.2830	0.2906	0.0741	0.2588	0.2604		0.2564		
**Nearable**										
	Withings Sleep Tracking Mat (n=75)	0.4921	0.5007	0.2455	0.4496	0.4205		0.4837		
	Google Nest Hub 2 (n=33)	0.4121	0.4089	0.0644	0.3009	0.2676		0.3299		
	Amazon Halo Rise (n=28)	0.6634	0.6706	0.4807	0.6242	0.6231		0.6031		
**Wearable**										
	Google Pixel Watch (n=30)	0.6355	0.6143	0.4044	0.5669	0.5381		0.5925		
	Galaxy Watch 5 (n=22)	0.6494	0.6499	0.4177	0.5761	0.6261		0.5651		
	Fitbit Sense 2 (n=26)	0.6464	0.6296	0.4185	0.5814	0.5130		0.6268		
	Apple Watch 8 (n=26)	0.5640	0.5731	0.2976	0.4910	0.5436		0.4203		
	Oura Ring 3 (n=53)	0.5427	0.5518	0.3492	0.5186	0.5187		0.5211		

^a^SNUBH: Seoul National University Bundang Hospital.

^b^CLC: Clionic Life Center.

^c^The number in parenthesis indicates the number of participants tested with each device.

^d^Top-performing consumer sleep tracker.

### Epoch-by-Epoch Analysis: Performance According to Sleep Stages

The performance of various sleep trackers across different sleep stages is presented in Table 3. For the wake and REM stages, SleepRoutine (airable) achieved the highest macro F1 scores of 0.7065 and 0.7596, respectively. These scores substantially surpassed those of the second-best tracker, Amazon Halo Rise (nearable), by a margin of 0.1098 for the wake stage and 0.0313 for the REM stage. For the deep stage, Google Pixel Watch and Fitbit Sense 2, which are wearables, exhibited superior performance with macro F1 scores of 0.5933 and 0.5564, respectively. Google Pixel Watch achieved the highest performance with a substantial margin. It surpassed Fitbit Sense 2 by a margin of 0.0368 and outpaced SleepRoutine, which was the sleep tracker with the third highest score, with an even larger margin of 0.0567. For the light stage, an array of sleep trackers, including 3 wearables (Google Pixel Watch, Galaxy Watch 5, and Fitbit Sense 2), 1 nearable (Amazon Halo Rise), and 1 airable (SleepRoutine), demonstrated similarly high levels of performance, with a macro F1 score ranging from 0.7142 to 0.7436. Additional detailed assessments of sleep stage performance, including accuracy, weighted F1, and area under the receiver operating characteristic curve metrics, are presented in Multimedia Appendices 3-6.

**Figure 3:**
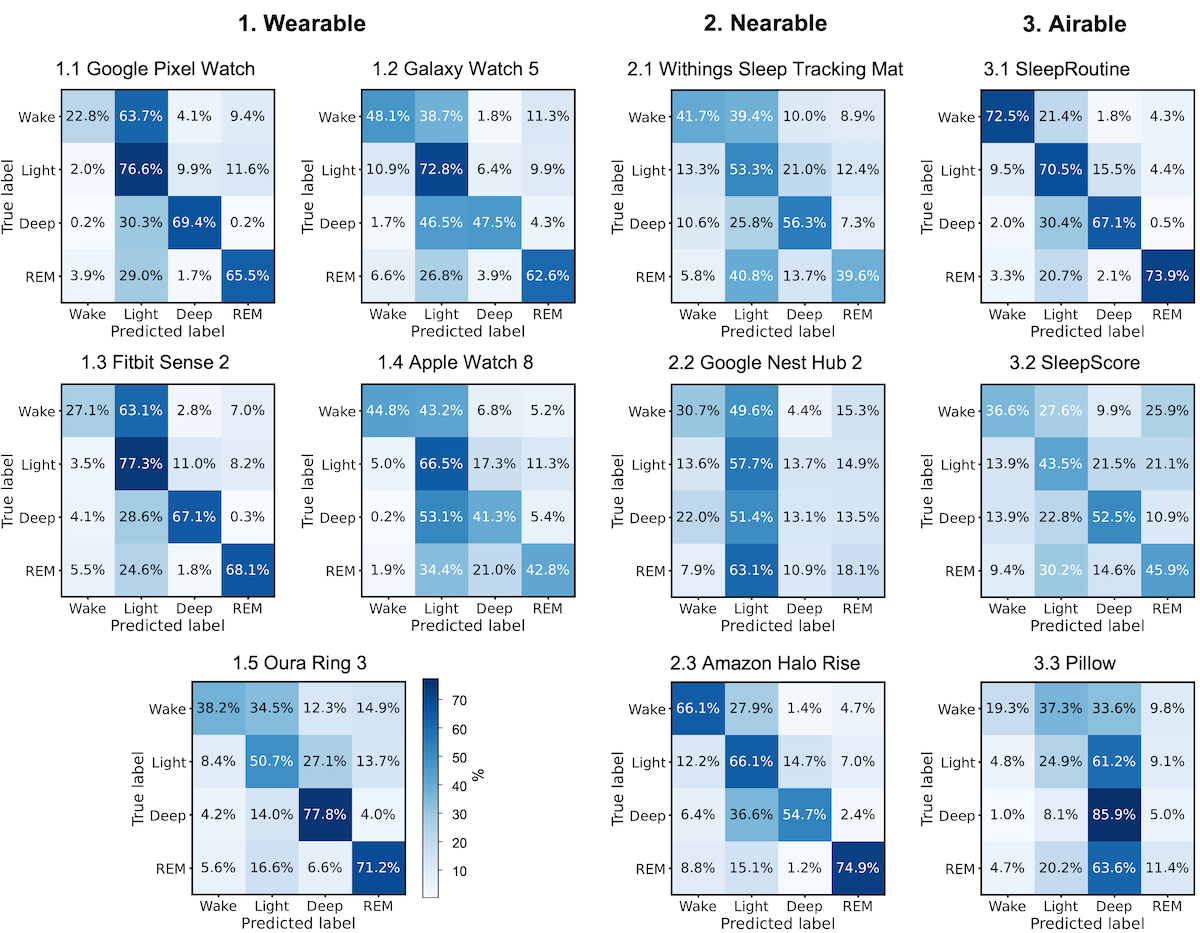
Normalized confusion matrices for 11 consumer sleep trackers (CSTs). Four-stage sleep classification confusion matrices comparing CSTs. Each row in the confusion matrix is the sleep stage annotated by polysomnography, while each column represents the sleep stage annotated by the CST. REM: rapid eye movement.

**Figure 4:**
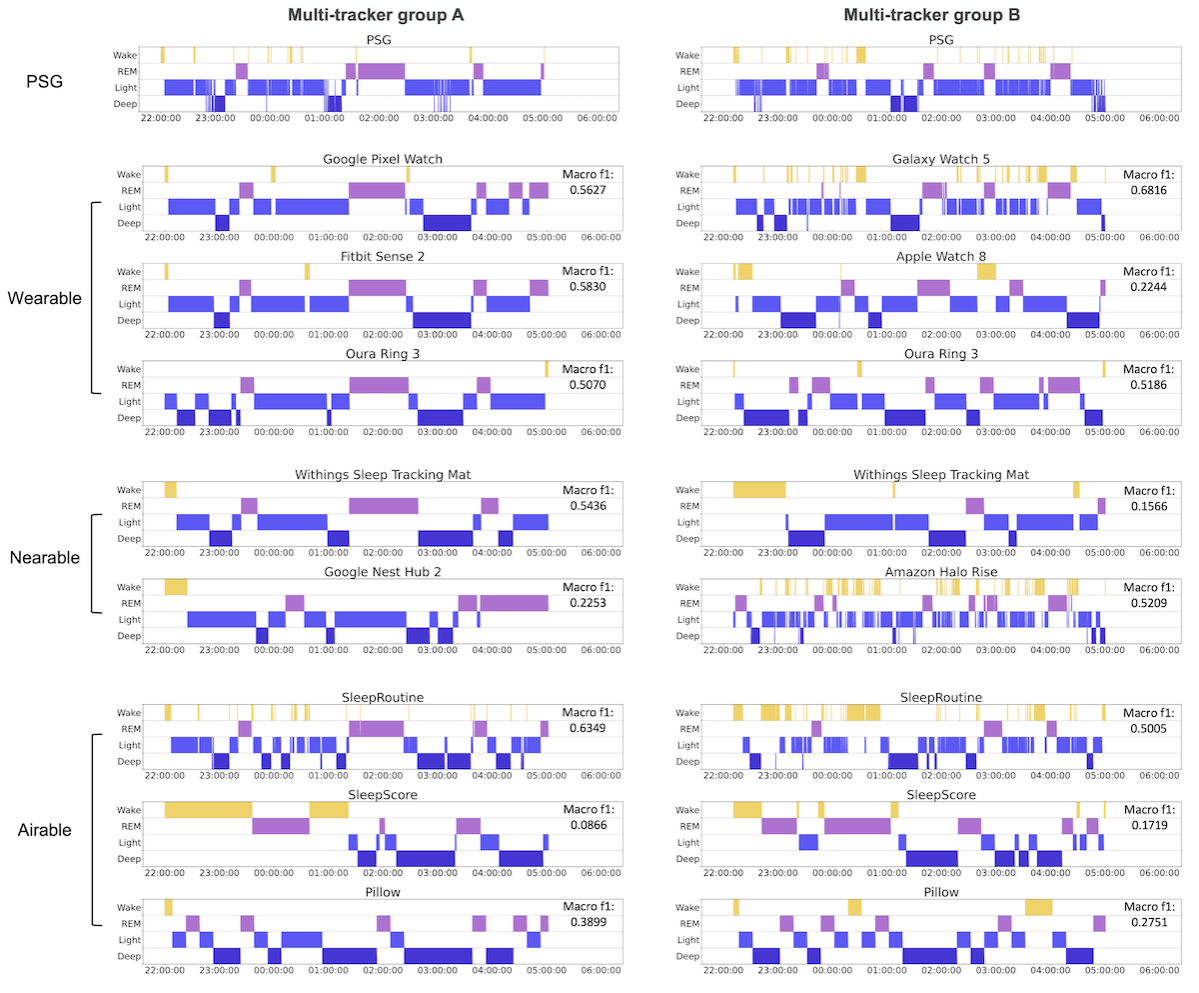
Sample hypnograms of 11 consumer sleep trackers (CSTs) involving 2 subjects in different groups. Hypnogram samples for each CST were selected based on the last measured subjects in multi-tracker group A (female; age, 35 years; BMI, 30.1; apnea-hypopnea index [AHI], 2.9) and multi-tracker group B (female; age, 26 years; BMI, 20; AHI, 3.5). PSG, polysomnography.

Figure 3 presents the confusion matrices for the sleep stages of the 11 CSTs, providing a clear visual representation of prediction biases and misclassification. Multimedia Appendix 7 presents the mean and variance of predicted values across participants. Analysis of the average tendencies across all devices revealed a prediction bias toward the light sleep stage. Google Nest Hub 2 (nearable) showed the largest bias toward the light stage among all the devices. Unlike other devices, Pillow (airable) was highly biased toward the deep stage, predicting 59% of epochs as deep, whereas only 10.8% of epochs were deep based on the results of polysomnography. The confusion matrices also revealed distinct patterns of misclassification in sleep stage prediction for device types. Wearables primarily misclassified wake as light, while nearables strongly misclassified REM as light. Airables, on the other hand, demonstrated a relatively higher frequency of confusion between the light and deep stages. Figure 4 presents a comparison of hypnograms illustrating the epoch-by-epoch agreement at the individual level, which facilitated the evaluation of agreement between CSTs and polysomnography in a time-series format. Additional hypnograms are presented in Multimedia Appendix 8.

Regarding Figure 4, the division of groups was necessary owing to the limited number of watches worn simultaneously, as explained in the Methods section. As 9 devices were used simultaneously for each subject, the hypnograms for each device are presented, with the polysomnography result displayed at the top. As shown in Figure 4, SleepRoutine, Amazon Halo Rise, and Galaxy Watch 5 exhibited more frequent transition of stages and predicted wake in the middle of sleep more frequently, resulting in better estimation of WASO, as shown in the analysis of sleep parameters.

**Table 3:** Epoch-by-epoch agreement: classification for detecting individual sleep stage.

Variable			Wake stage^a^							Light stage^a^							Deep stage^a^							REM^b^ stage^a^				
			F1		Sensitivity		Specificity		F1			Sensitivity		Specificity		F1			Sensitivity		Specificity		F1			Sensitivity		Specificity
**Airable**																												
	SleepRoutine (n=67)^c^	0.7065^d^		0.7246^d^		0.9269		0.7436^d^			0.7054		0.7665^d^		0.5355			0.6712		0.8973		0.7596^d^			0.7394		0.9609^d^	
	SleepScore (n=38)	0.4057		0.3665		0.8696		0.5147			0.4355		0.7272		0.3574			0.5247		0.8264		0.3418			0.4587		0.7895	
	Pillow (n=74)	0.2828		0.1934		0.9572		0.3409			0.2490		0.7534		0.2673			0.8594^d^		0.4449		0.1440			0.1140		0.9126	
**Nearable**																												
	Withings Sleep Tracking Mat (n=75)	0.4419		0.4172		0.8854		0.5764			0.5328		0.6336		0.3800			0.5633		0.8270		0.4001			0.3964		0.8906	
	Google Nest Hub 2 (n=33)	0.3296		0.3068		0.8649		0.5619			0.5772		0.4518		0.1245			0.1308		0.8883		0.1876			0.1805		0.8514	
	Amazon Halo Rise (n=28)	0.5967		0.6612		0.8921		0.7142			0.6609		0.7484		0.4575			0.5467		0.9018		0.7283			0.7490^d^		0.9401	
**Wearable**																												
	Google Pixel Watch (n=30)	0.3456		0.2277		0.9784^d^		0.7150			0.7657		0.5620		0.5922^d^			0.6937		0.9290		0.6146			0.6548		0.9029	
	Galaxy Watch 5 (n=22)	0.4755		0.4814		0.9104		0.7346			0.7280		0.6412		0.4963			0.4752		0.9481^d^		0.5982			0.6265		0.9058	
	Fitbit Sense 2 (n=26)	0.3807		0.2714		0.9602		0.7262			0.7734^d^		0.5727		0.5564			0.6710		0.9247		0.6623			0.6812		0.9297	
	Apple Watch 8 (n=26)	0.5493		0.4481		0.9624		0.6680			0.6649		0.5737		0.3073			0.4130		0.8412		0.4394			0.4276		0.9070	
	Oura Ring 3 (n=53)	0.4527		0.3822		0.9264		0.5953			0.5072		0.7630		0.4272			0.7784		0.7974		0.5993			0.7118		0.8716	

^a^Individual sleep stage classification was used to categorize each class and the remaining classes.

^b^REM: rapid eye movement.

^c^The number in parenthesis indicates the number of participants tested with each device.

^d^Top-performing consumer sleep tracker.

### Sleep Measure Analysis

**Figure 5:**
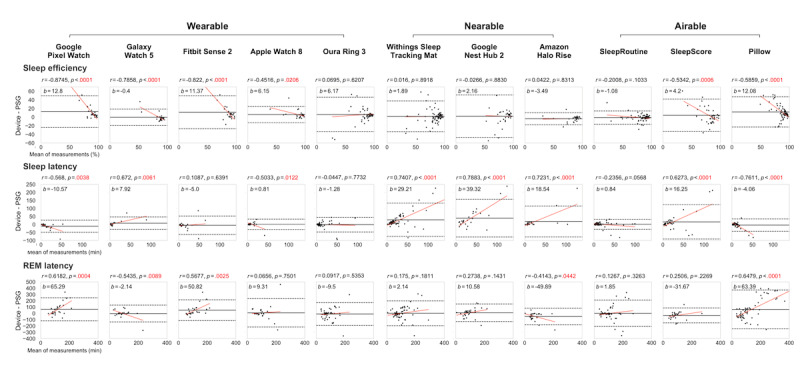
Bland-Altman plots of consumer sleep trackers (CSTs) and polysomnography (PSG) for sleep efficiency, sleep latency, and rapid eye movement (REM) latency measurements. The plots present the mean bias (middle horizontal black solid line), and upper (upper horizontal black dashed line) and lower (lower horizontal black dashed line) limits of agreement. In the figure, “b” represents bias and “r” denotes the Pearson correlation coefficient between the mean of measurements and the difference between the CSTs and PSG. The correlation coefficient is displayed along with its corresponding *P* value. The red line indicates the estimated linear regression line.

Figure 5 presents the Bland-Altman plots of CSTs, illustrating the performance of sleep measurements, including sleep efficiency, sleep latency, and REM latency, when compared with polysomnography. The average value of polysomnography sleep efficiency ranged from 77.57% to 86.05%, while the bias for each CST varied from −3.4909 percentage points (Amazon Halo Rise) to 12.8035 percentage points (Google Pixel Watch). Polysomnography values for sleep latency ranged from 10.80 minutes to 19.80 minutes, with CST biases ranging from −0.81 minutes (Apple Watch 8) to 39.42 minutes (Google Nest Hub 2). Polysomnography values for REM latency ranged from 87.00 minutes to 112.20 minutes, with CST biases ranging from −49.89 minutes (Amazon Halo Rise) to 65.29 minutes (Google Pixel Watch). The devices demonstrated distinct and best performances for each sleep metric. In terms of sleep efficiency, Galaxy Watch 5 (wearable) achieved a minimal bias of −0.4%. In the case of estimation of sleep latency, Apple Watch 8 (wearable) exhibited a bias of 0.81 minutes. Lastly, SleepRoutine (airable) demonstrated the best performance for REM latency with a bias of 1.85 minutes. The proportional bias, presented as “r” in Figure 5, indicates how consistent the mean bias was regardless of the sleep measure. Oura Ring and SleepRoutine showed no proportional bias (ie, no significant correlation in the Bland-Altman plot) for any sleep measure. The difference in mean values between polysomnography and each CST for each sleep measure is described in Multimedia Appendix 9. Additional information is provided in Multimedia Appendix 10.

### Subgroup Analysis

Subgroup analyses were conducted for all devices, considering factors, including sex assigned at birth, AHI, sleep efficiency, and BMI. The macro F1 scores for each subgroup are presented in Table 4. Multimedia Appendix 11 presents the subgroup analysis results of epoch-by-epoch agreement for the AHI. The average performance of CSTs showed a comprehensive relationship between sleep tracker performance and these parameters. In terms of BMI, the average macro F1 score was 0.5043 for individuals with a BMI of ≤25 kg/m^2^, whereas it dropped to 0.4790 for those with a BMI of >25 kg/m^2^, indicating a gap of 0.0253. Similarly, for sleep efficiency, the scores were 0.4757 for individuals with a sleep efficiency of ≤85% and 0.4902 for those with a sleep efficiency of >85%, with a difference of 0.0145. In the case of the AHI, the scores were 0.4905 for an AHI of ≤15 and 0.5024 for an AHI of >15, resulting in a difference of 0.0119. In contrast, the difference between male and female subgroups was minimal, with a macro F1 score of 0.4926 for males and 0.4932 for females, resulting in a negligible difference of 0.0006. In each subgroup, the highest variations were observed with the airable SleepScore for AHI (difference: 0.0929), the nearable Google Pixel Watch for sleep efficiency (difference: 0.1067), the wearable Galaxy Watch 5 for BMI (difference: 0.0785), and the airable SleepScore for sex assigned at birth (difference: 0.0872). Multimedia Appendices 12 and 13 present the subgroup analysis results of epoch-by-epoch agreement in the institutions. Additionally, Multimedia Appendices 14 and 15 provide an overview of the average macro F1 scores individually calculated for each participant.

**Table 4:** Epoch-by-epoch agreement: subgroup analysis of the apnea-hypopnea index and demographic characteristics.

Variable		Apnea-hypopnea index		Sleep efficiency		BMI		Gender	
		≤15	>15	≤85%	>85%	≤25	>25	Male	Female
**Airable**									
	SleepRoutine (n=67)^a^	0.6536^b^	0.7320^b^	0.6971^b^	0.6490^b^	0.6840^b^	0.6889^b^	0.7137^b^	0.6568^b^
	SleepScore (n=38)	0.3636	0.4565	0.4107	0.3808	0.4118	0.3937	0.4431	0.3559
	Pillow (n=74)	0.2602	0.2567	0.2472	0.2567	0.2601	0.2548	0.2670	0.2446
**Nearable**									
	Withings Sleep Tracking Mat (n=75)	0.4644	0.4225	0.3766	0.4653	0.4760	0.3964	0.4587	0.4355
	Google Nest Hub 2 (n=33)	0.3000	0.3059	0.3115	0.2762	0.3209	0.2517	0.2889	0.3059
	Amazon Halo Rise (n=28)	0.6160	0.6389	0.6297	0.5857	0.6414	0.5801	0.6075	0.6491
**Wearable**									
	Google Pixel Watch (n=30)	0.5670	0.5626	0.5035	0.6102	0.5653	0.5791	0.5235	0.5956
	Galaxy Watch 5 (n=22)	0.5701	0.5790	0.6029	0.5547	0.5521	0.6306	0.5655	0.5867
	Fitbit Sense 2 (n=26)	0.5839	0.5753	0.5325	0.6090	0.5910	0.5541	0.5320	0.6129
	Apple Watch 8 (n=26)	0.4861	0.4950	0.4326	0.4804	0.5093	0.4561	0.5263	0.4414
	Oura Ring 3 (n=53)	0.5302	0.5021	0.4882	0.5245	0.5354	0.4830	0.4926	0.5405

^a^The number in parenthesis indicates the number of participants tested with each device.

^b^Top-performing consumer sleep trackers.

## Discussion

### Principal Findings

**Figure 6:**
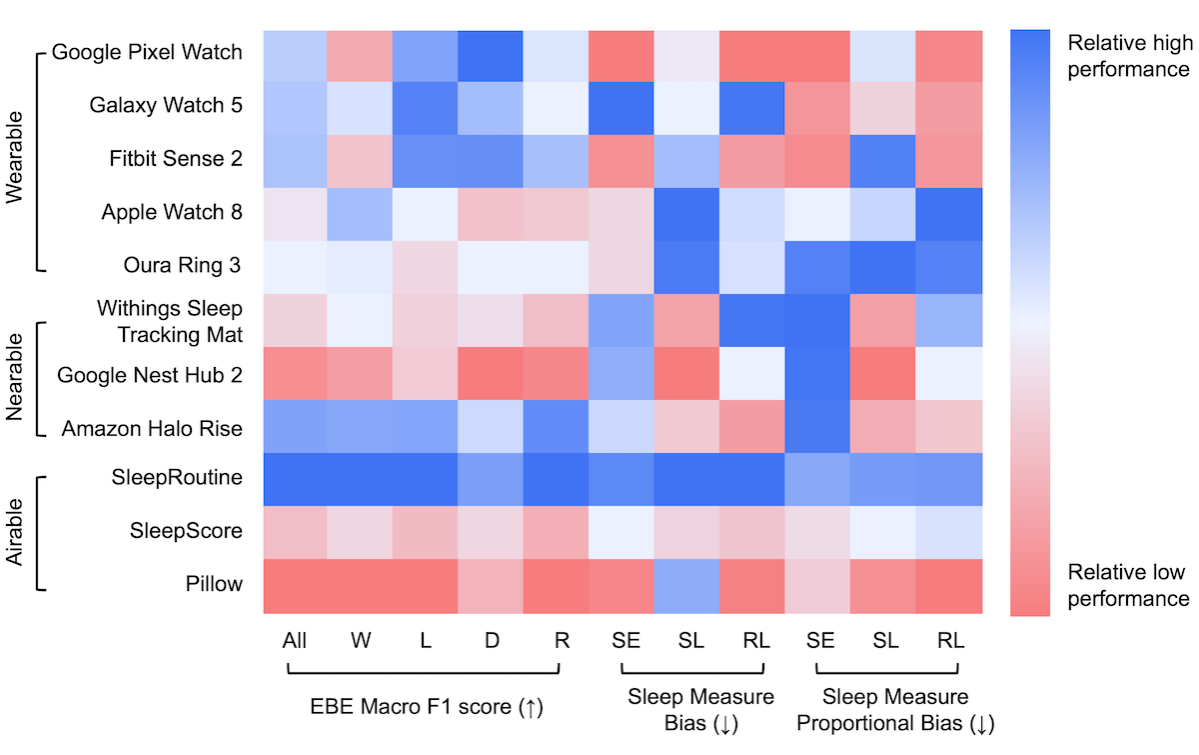
Relative performance rank heatmap of 11 consumer sleep trackers (CSTs). Heatmap of the relative performance of sleep stage and classification of sleep measures normalized to the highest and lowest macro F1 values in each CST. D: deep; EBE: epoch-by-epoch agreement; L: light; R: rapid eye movement; RL: rapid eye movement latency; SE: sleep efficiency; SL: sleep latency; W: wake.

We conducted an extensive analysis of 11 CSTs involving 75 subjects, which, to the best of our knowledge, represents the largest number of devices simultaneously evaluated in the literature [3,4,10,11,13,18]. The findings are illustrated in Figure 6, which presents the relative performances of the 11 CSTs in estimating sleep stages and sleep measures. Our findings revealed that Google Pixel Watch, Galaxy Watch 5, and Fitbit Sense 2 demonstrated competitive performance among wearables, while Amazon Halo Rise and SleepRoutine stood out among nearables and airables, respectively.

### Wearables

Wearables, including watch and ring-type sleep trackers, represent the most prevalent CSTs in the market [23,24]. They employ photoplethysmography sensors and accelerometer sensors to measure cardiac activity (eg, heart rate variability) and body movements. Given their reliance on similar biosignals for sleep tracking, wearables exhibit consistent patterns in estimating sleep stages. First, most wearables generally overestimate sleep by misclassifying wake stages, leading to a substantial negative proportional bias in estimating sleep efficiency, which results in worse performance for individuals with low sleep efficiency (Figure 5). This bias was specifically observed when actigraphy was used to measure sleep efficiency and WASO [5,11]. This can be attributed to the dependence of actigraphy and wearables on body movement to determine sleep-wake states. Insomniacs often lie still in bed while trying to sleep, even though they are actually awake [24]. As a result, these periods of wakefulness can be misinterpreted as sleep. Nevertheless, Oura Ring showed negligible proportional bias, potentially owing to its use of additional features, such as body temperature and circadian rhythm, for sleep staging [6]. Second, wearables comprising the top 3 CSTs demonstrated substantial alignment in the classification of deep stages. In particular, the results from Oura Ring 3 and Fitbit Sense 2 in this study showed improved accuracy in sleep stage detection compared to previous studies that focused on earlier versions of Oura Ring and Fitbit in assessing the accuracy of wearable sleep evaluations [25,26]. Thus, wearables may facilitate accurate detection of different stages of deep sleep, given their unique association with autonomic nervous system stabilization. Heart rate variability, a key indicator of autonomic nervous system activity, can be directly measured by photoplethysmography sensors [27]. Therefore, wearables are effective in monitoring deep sleep stages.

### Nearables

Nearables, encompassing pad and motion sensor-type sleep trackers, use overall body movements and respiratory efforts (thoracic and abdominal) for sleep monitoring. Similar to wearables, nearables also exhibit aligned tendencies. First, all nearables tend to overestimate sleep onset latency, resulting in a significant mean bias (29.02 minutes for nearables, −2.71 minutes for wearables, and 4.34 minutes for airables) and a significant positive proportional bias in sleep latency measurement. This indicates that nearables may overestimate sleep latency, particularly in individuals with prolonged sleep latency. During extended periods of attempting to fall asleep in bed, users may experience increased restlessness and movement, which makes it challenging for nearables to estimate the sleep stage using radar-like sensors [28]. Second, unlike wearables, nearables demonstrated the least sensitivity in deep stage classification (as shown in Figure 3). Distinguishing stages of deep sleep from light sleep based on variations in respiratory patterns requires precise monitoring of respiratory activity. However, the radar-like sensors employed by nearables, while efficient at detecting larger body movements, have difficulty capturing smaller fidgeting movements, which represent a challenge in accurately identifying the stage of deep sleep.

### Airables

Airables excel in terms of their accessibility by not requiring the purchase of additional hardware. However, their clinical validation is not well-established as noted in previous studies up until 2022, which highlighted the limited agreement between airables and polysomnography as a notable limitation [25,29]. In this study, our aim was to validate the latest airable CSTs. One distinguishing feature of airable CSTs is their use of diverse sensor types, and the variation in performance is substantially influenced by the specific sensor type and accompanying algorithm. Thus, we chose 3 types of airable CSTs considering diversity (microphone, ultrasound, and accelerometer-based applications). These methodological distinctions contribute to pronounced variations in the determination of sleep stage. Pillow requires placement on the mattress and uses the smartphone’s accelerometer sensor to detect user movements through the mattress. Notably, Pillow showed a prediction bias toward the deep stage, suggesting that movement information during sleep was insufficient for the accurate determination of sleep stage. SleepScore uses a sonar biomotion sensor and directs the smartphone’s speaker toward the chest area to emit ultrasonic signals above 18 kHz, tracking thoracic respiratory effort. Depending on the biosignal used, SleepScore shows similar tendencies with nearables, demonstrating a substantial mean bias and positive proportional bias in estimating sleep onset latency. SleepRoutine analyzes the sound recorded during sleep [2]. Sleep sounds provide a wealth of sleep-related information, including changes in breathing regularity linked to autonomic nervous system stabilization, changes in breathing sound characteristics (such as tone, pitch, and amplitude) due to altered respiratory muscle tension, and noise from body movements. Among all CSTs, SleepRoutine exhibited the highest accuracy in predicting the wake and REM stages.

### REM Sleep Stage Estimation Performance

REM was the stage where most CSTs demonstrated relatively higher agreement with polysomnography compared with other stages. Among the top 5 CSTs with the highest macro F1 scores (SleepRoutine, Amazon Halo Rise, Fitbit Sense 2, Galaxy Watch 5, and Google Pixel Watch), the REM stage showed a substantially higher average F1 score of 0.672, compared with 0.501 for wake and 0.528 for deep sleep. This can be attributed to the unique characteristics of REM sleep, which include increased irregularity in heart rate and breathing, minimal muscle movement, and rapid variations in blood pressure and body temperature [30]. These features allow easy detection of different types of biosignals and accurate classification of REM sleep.

### Cost-Effectiveness

We evaluated the costs of 11 sleep tracking technologies based on the costs analyzed in Multimedia Appendix 16. Wearables, with an average price of US $386, offer a wide range of functions, including messaging and various apps, beyond sleep tracking. Oura Ring, while lacking these supplementary features, provides a broad spectrum of health tracking functions. Nearables, with an average price of US $123, include a variety of features across different models. Google Nest Hub and Amazon Halo Rise offer extra features, such as an IoT hub and wake-up light, whereas Withings Sleep Mat is exclusively designed for sleep tracking. Airables, which are app-based technologies, harness smartphone sensors for sleep tracking, requiring only a subscription fee of US $53 and no additional hardware. This economical and flexible option, which can be easily canceled, represents a cost-effective solution for sleep tracking.

### Standardized Validation and Data Transparency

Standardized methods of validation and data transparency are crucial for comparing sleep trackers [31], particularly due to the increasing use of deep learning algorithms whose inner workings are often opaque. In our study, we adhered to established frameworks for standardized validation [15,32], while also conducting multi-center evaluations based on diverse demographic factors. Regarding data transparency, we provided comprehensive details of validation; however, obtaining access to the training data of each CST was challenging. Transparency in both training and validation data is essential for building trustworthy artificial intelligence models and can also contribute to a better understanding of CSTs [33].

### Limitations

It is important to note the limitations of our study. First, data collection rates significantly varied between the 2 institutions as the study was independently implemented. Issues, such as battery management, account management, and human errors, resulted in data omissions. Second, demographic differences were detected between the institutions, including disparities in time spent in bed and total sleep time. Operational issues led to slightly earlier waking of participants in CLC. Third, this study focused solely on the Korean population, with limited ability to analyze performance differences among various races. Future studies should incorporate multiracial comparisons and evaluate CST performance across diverse home environments for realistic assessments.

### Conclusions

Our study represents a comprehensive and comparative analysis of 11 CSTs and their accuracy in tracking sleep in a sleep lab setting. The objective of this study was to gain insights into the performance and capabilities of these CSTs. Personalized sleep health management is necessary to enable individuals to make informed choices for monitoring and improving sleep quality. Further, our findings emphasize the importance of understanding the characteristics and limitations of these devices. It lays the foundation for guiding the development of sleep trackers in the future. Accordingly, future studies should focus on developing accurate sleep stage classification systems by integrating different types of biosignals in a home environment.

## Appendix

Multimedia Appendix 1 The number of observations in each institution.

Multimedia Appendix 2 Comparative analysis of participant demographics across institutions (multi-tracker group A vs multi-tracker group B).

Multimedia Appendix 3 Epoch-by-epoch agreement: wake stage classification.

Multimedia Appendix 4 Epoch-by-epoch agreement: light stage classification.

Multimedia Appendix 5 Epoch-by-epoch agreement: deep stage classification.

Multimedia Appendix 6 Epoch-by-epoch agreement: rapid eye movement stage classification.

Multimedia Appendix 7 Normalized confusion matrices: mean and variance of predicted values across participants.

Multimedia Appendix 8 Additional hypnogram examples for 11 consumer sleep trackers of 2 subjects in different groups.

Multimedia Appendix 9 Comparison of sleep parameters with polysomnography.

Multimedia Appendix 10 Supplementary results of the sleep measure analysis.

Multimedia Appendix 11 Epoch-by-epoch agreement: subgroup analysis of the apnea-hypopnea index.

Multimedia Appendix 12 Epoch-by-epoch agreement: subgroup analysis of the apnea-hypopnea index and demographic characteristics in Seoul National University Bundang Hospital.

Multimedia Appendix 13 Epoch-by-epoch agreement: subgroup analysis of the apnea-hypopnea index and demographic characteristics in Clionic Lifecare Clinic.

Multimedia Appendix 14 Group-averaged macro F1 scores: subgroup analysis of the apnea-hypopnea index and demographic characteristics in Seoul National University Bundang Hospital.

Multimedia Appendix 15 Group-averaged macro F1 scores: subgroup analysis of the apnea-hypopnea index and demographic characteristics in Clionic Lifecare Clinic.

Multimedia Appendix 16 Cost-effectiveness of consumer sleep trackers.

## Abbreviations

AHI: apnea-hypopnea index
CLC: Clionic Lifecare Clinic
CST: consumer sleep tracker
REM: rapid eye movement
SNUBH: Seoul National University Bundang Hospital
WASO: wake after sleep onset

## Data Availability

Access to data sets from Seoul National University Bundang Hospital and Clionic Lifecare Clinic, used with permission for this study, should be requested directly from these institutions. Subject to ethical approval from the institutional review boards, the corresponding author agrees to share deidentified individual participant data, the study protocol, and the statistical analysis plan with academic researchers following completion of a data use agreement. Proposals should be directed to the corresponding author. All experimental and implementation details that can be shared are described in detail in this paper and the multimedia appendices.

## References

[1] Watson N, Badr M, Belenky G, Bliwise D, Buxton O, Buysse D, Dinges D, Gangwisch J, Grandner M, Kushida C, Malhotra R, Martin J, Patel S, Quan S, Tasali E (2015). Recommended amount of sleep for a healthy adult: a Joint Consensus Statement of the American Academy of Sleep Medicine and Sleep Research Society. Sleep.

[2] Tran HH, Hong JK, Jang H, Jung J, Kim J, Hong J, Lee M, Kim J, Kushida CA, Lee D, Kim D, Yoon I (2023). Prediction of sleep stages via deep learning using smartphone audio recordings in home environments: model development and validation. J Med Internet Res.

[3] Fino E, Plazzi G, Filardi M, Marzocchi M, Pizza F, Vandi S, Mazzetti M (2020). (Not so) Smart sleep tracking through the phone: findings from a polysomnography study testing the reliability of four sleep applications. J Sleep Res.

[4] Chinoy ED, Cuellar JA, Jameson JT, Markwald RR (2022). Performance of four commercial wearable sleep-tracking devices tested under unrestricted conditions at home in healthy young adults. Nat Sci Sleep.

[5] Marino M, Li Y, Rueschman MN, Winkelman JW, Ellenbogen JM, Solet JM, Dulin H, Berkman LF, Buxton OM (2013). Measuring sleep: accuracy, sensitivity, and specificity of wrist actigraphy compared to polysomnography. Sleep.

[6] Altini M, Kinnunen H (2021). The Promise of Sleep: A Multi-Sensor Approach for Accurate Sleep Stage Detection Using the Oura Ring. Sensors (Basel).

[7] Hong JK, Lee T, Delos Reyes RD, Hong J, Tran HH, Lee D, Jung J, Yoon I (2021). Confidence-based framework using deep learning for automated sleep stage scoring. Nat Sci Sleep.

[8] Fino E, Mazzetti M (2019). Monitoring healthy and disturbed sleep through smartphone applications: a review of experimental evidence. Sleep Breath.

[9] Tuominen J, Peltola K, Saaresranta T, Valli K (2019). Sleep Parameter Assessment Accuracy of a Consumer Home Sleep Monitoring Ballistocardiograph Beddit Sleep Tracker: A Validation Study. J Clin Sleep Med.

[10] Mantua J, Gravel N, Spencer RMC (2016). Reliability of sleep measures from four personal health monitoring devices compared to research-based actigraphy and polysomnography. Sensors (Basel).

[11] Chinoy ED, Cuellar JA, Huwa KE, Jameson JT, Watson CH, Bessman SC, Hirsch DA, Cooper AD, Drummond SPA, Markwald RR (2021). Performance of seven consumer sleep-tracking devices compared with polysomnography. Sleep.

[12] Klier K, Wagner M (2022). Agreement of Sleep Measures-A Comparison between a Sleep Diary and Three Consumer Wearable Devices. Sensors (Basel).

[13] Haghayegh S, Khoshnevis S, Smolensky MH, Diller KR, Castriotta RJ (2019). Accuracy of wristband fitbit models in assessing sleep: systematic review and meta-analysis. J Med Internet Res.

[14] Shcherbina A, Mattsson CM, Waggott D, Salisbury H, Christle JW, Hastie T, Wheeler MT, Ashley EA (2017). Accuracy in wrist-worn, sensor-based measurements of heart rate and energy expenditure in a diverse cohort. J Pers Med.

[15] Nguyen QNT, Le T, Huynh QBT, Setty A, Vo TV, Le TQ (2021). Validation framework for sleep stage scoring in wearable sleep trackers and monitors with polysomnography ground truth. Clocks Sleep.

[16] Menghini L, Yuksel D, Goldstone A, Baker FC, de Zambotti M (2021). Performance of Fitbit Charge 3 against polysomnography in measuring sleep in adolescent boys and girls. Chronobiol Int.

[17] Schade MM, Bauer CE, Murray BR, Gahan L, Doheny EP, Kilroy H, Zaffaroni A, Montgomery-Downs HE (2019). Sleep Validity of a Non-Contact Bedside Movement and Respiration-Sensing Device. J Clin Sleep Med.

[18] Kolla BP, Mansukhani S, Mansukhani MP (2016). Consumer sleep tracking devices: a review of mechanisms, validity and utility. Expert Rev Med Devices.

[19] Robbins R, Seixas A, Masters LW, Chanko N, Diaby F, Vieira D, Jean-Louis G (2019). Sleep tracking: A systematic review of the research using commercially available technology. Curr Sleep Med Rep.

[20] Mouritzen NJ, Larsen LH, Lauritzen MH, Kjær T (2020). Assessing the performance of a commercial multisensory sleep tracker. PLoS One.

[21] Ananth S (2021). Sleep apps: current limitations and challenges. Sleep Sci.

[22] Berry RB, Brooks R, Gamaldo CE, Harding SM, Marcus CL, Vaughn BV, Tangredi MM The AASM Manual for the Scoring of Sleep and Associated Events: Rules, Terminology and Technical Specifications. Neumosur.

[23] (2023). Global Market Insights.

[24] Schutte-Rodin S, Broch L, Buysse D, Dorsey C, Sateia M (2008). Clinical guideline for the evaluation and management of chronic insomnia in adults. J Clin Sleep Med.

[25] de Zambotti M, Cellini N, Goldstone A, Colrain IM, Baker FC (2019). Wearable sleep technology in clinical and research settings. Med Sci Sports Exerc.

[26] de Zambotti M, Goldstone A, Claudatos S, Colrain IM, Baker FC (2018). A validation study of Fitbit Charge 2™ compared with polysomnography in adults. Chronobiol Int.

[27] Vanoli E, Adamson PB, Pinna GD, Lazzara R, Orr WC, Ba-Lin (1995). Heart rate variability during specific sleep stages. A comparison of healthy subjects with patients after myocardial infarction. Circulation.

[28] Quick Installation Guide - Withings Sleep Analyzer. Withings.

[29] Ibáñez V, Silva J, Navarro E, Cauli O (2019). Sleep assessment devices: types, market analysis, and a critical view on accuracy and validation. Expert Rev Med Devices.

[30] Shrivastava D, Jung S, Saadat M, Sirohi R, Crewson K (2014). How to interpret the results of a sleep study. J Community Hosp Intern Med Perspect.

[31] Lehmann CU, Miller MR (2004). Standardization and the practice of medicine. J Perinatol.

[32] Menghini L, Cellini N, Goldstone A, Baker FC, de Zambotti M (2021). A standardized framework for testing the performance of sleep-tracking technology: step-by-step guidelines and open-source code. Sleep.

[33] Good Machine Learning Practice for Medical Device Development: Guiding Principles. US FDA.

