# Supplementary material for: Accuracy of 11 Wearable, Nearable, and Airable Consumer Sleep Trackers: Prospective Multicenter Validation Study
Source: JMIR Mhealth Uhealth. 2023 Nov 2;11:e50983. doi: 10.2196/50983 (PMC10654909; doi:10.2196/50983)
Supplement: Multimedia Appendix 1 [file mhealth_v11i1e50983_app1.pdf]

Multimedia Appendix 1. The number of observations in each institution.

Device	SNUBH (n)	SNUBH (%)	CLC (n)	CLC (%)
**Airable**				
SleepRoutine (67)	29	78.38	38	100.00
SleepScore (38)	26	70.27	12	31.58
Pillow (74)	37	100.00	37	97.37
**Nearable**				
Withings Sleep Tracking Mat (75)	37	100.00	38	100.00
Google Nest Hub 2 (33)	14	77.78	19	100.00
Amazon Halo Rise (28)	12	63.16	16	84.21
**Wearable**				
Google Pixel Watch (30)	12	66.67	18	94.74
Galaxy Watch 5 (22)	3	15.79	19	100.00
Fitbit Sense 2 (26)	9	50.00	17	89.47
Apple Watch 8 (26)	8	42.11	18	94.74
Oura Ring 3 (53)	22	59.46	31	81.58

The numbers and percentages in the table represent the number of participants measured for each CST and the success rate of data collection at the two institutions.
