# Supplementary material for: Accuracy of 11 Wearable, Nearable, and Airable Consumer Sleep Trackers: Prospective Multicenter Validation Study
Source: JMIR Mhealth Uhealth. 2023 Nov 2;11:e50983. doi: 10.2196/50983 (PMC10654909; doi:10.2196/50983)
Supplement: Multimedia Appendix 2 [file mhealth_v11i1e50983_app2.pdf]

Multimedia Appendix 2. Comparative analysis of participant demographics across institutions (multi-tracker group A vs multi-tracker group B).

	SNUBH			CLS		
	**Multi-tracker group A**	**Multi-tracker group B**	**P**	**Multi-tracker group A**	**Multi-tracker group B**	**P**
Number of participants	18	19		19	19	
Male, % (n)	72.22(13)	73.68(14)	-	10.53 (2)	52.63 (10)	-
Age, years	55.0±12.41	52.05±11.67	0.4615	35.0±10.58	32.89±4.45	0.4291
Body mass index, kg/m²	24.12±4.35	25.13±3.85	0.4615	22.71±3.91	23.64±4.1	0.4786
Time in bed, hours	8.02±0.22	8.01±0.39	0.9371	6.48±0.69	6.5±0.65	0.9535
Total sleep time, hours	6.34±1.16	6.19±1.26	0.7133	5.21±1.49	5.59±1.17	0.3978
Sleep latency, hours	0.26±0.38	0.28±0.39	0.8650	0.32±0.38	0.2±0.37	0.3544
Wake After Sleep Onset, hours	1.42±1.09	1.54±1.35	0.7722	0.95±1.45	0.71±1.0	0.5484
Sleep efficiency, %	79.15±14.74	77.69±16.63	0.7809	80.88±21.64	86.19±16.34	0.3989
Apnea hypopnea index	28.17±24.48	25.04±24.59	0.7010	9.61±12.4	10.43±9.7	0.8214

Percentage (%) of total population (number of participants), mean ± standard deviation. All p-values were obtained using two-sample independent t-tests. Abbreviations: SNUBH Seoul National Bundang Hospital, CLC Clionic Life Center.
