# Supplementary material for: Accuracy of 11 Wearable, Nearable, and Airable Consumer Sleep Trackers: Prospective Multicenter Validation Study
Source: JMIR Mhealth Uhealth. 2023 Nov 2;11:e50983. doi: 10.2196/50983 (PMC10654909; doi:10.2196/50983)
Supplement: Multimedia Appendix 3 [file mhealth_v11i1e50983_app3.pdf]

Multimedia Appendix 3. Epoch-by-epoch agreement: wake stage classification.

Device	Sensitivity	Specificity	Accuracy	F1 score	Weighted F1	AUROC
**Airable**						
SleepRoutine (67)	**0.7246**	0.9269	**0.8898**	**0.7065**	**0.8909**	**0.8257**
SleepScore (38)	0.3665	0.8696	0.7546	0.4057	0.7449	0.6180
Pillow (74)	0.1934	0.9572	0.8066	0.2828	0.7688	0.5753
**Nearable**						
Withings Sleep Tracking Mat (75)	0.4172	0.8854	0.7938	0.4419	0.7890	0.6513
Google Nest Hub 2 (33)	0.3068	0.8649	0.7556	0.3296	0.7485	0.5858
Amazon Halo Rise (28)	0.6612	0.8921	0.8545	0.5967	0.8600	0.7767
**Wearable**						
Google Pixel Watch (30)	0.2277	**0.9784**	0.8329	0.3456	0.7959	0.6030
Galaxy Watch 5 (22)	0.4814	0.9104	0.8496	0.4755	0.8504	0.6959
Fitbit Sense 2 (26)	0.2714	0.9602	0.8189	0.3807	0.7887	0.6158
Apple Watch 8 (26)	0.4481	0.9624	0.8748	0.5493	0.8630	0.7052
Oura Ring 3 (53)	0.3822	0.9264	0.8209	0.4527	0.8076	0.6543

Wake stages were used to classify the wake class and the remaining classes. The top performing consumer sleep trackers are shown in bold. Abbreviation: AUROC, area under receiver operating characteristic curve.
