# Supplementary material for: Accuracy of 11 Wearable, Nearable, and Airable Consumer Sleep Trackers: Prospective Multicenter Validation Study
Source: JMIR Mhealth Uhealth. 2023 Nov 2;11:e50983. doi: 10.2196/50983 (PMC10654909; doi:10.2196/50983)
Supplement: Multimedia Appendix 4 [file mhealth_v11i1e50983_app4.pdf]

Multimedia Appendix 4. Epoch-by-epoch agreement: light stage classification.

Device	Sensitivity	Specificity	Accuracy	F1 score	Weighted F1	AUROC
**Airable**						
SleepRoutine (67)	0.7054	**0.7665**	**0.7330**	**0.7436**	**0.7336**	**0.7360**
SleepScore (38)	0.4355	0.7272	0.5769	0.5147	0.5681	0.5813
Pillow (74)	0.2490	0.7534	0.4822	0.3409	0.4485	0.5012
**Nearable**						
Withings Sleep Tracking Mat (75)	0.5328	0.6336	0.5795	0.5764	0.5793	0.5832
Google Nest Hub 2 (33)	0.5772	0.4518	0.5188	0.5619	0.5174	0.5145
Amazon Halo Rise (28)	0.6609	0.7484	0.6985	0.7142	0.6999	0.7047
**Wearable**						
Google Pixel Watch (30)	**0.7657**	0.5620	0.6716	0.7150	0.6677	0.6638
Galaxy Watch 5 (22)	0.7280	0.6412	0.6920	0.7346	0.6925	0.6846
Fitbit Sense 2 (26)	0.7734	0.5727	0.6821	0.7262	0.6784	0.6730
Apple Watch 8 (26)	0.6649	0.5737	0.6254	0.6680	0.6257	0.6193
Oura Ring 3 (53)	0.5072	0.7630	0.6233	0.5953	0.6191	0.6351

Light stage classification was used to for light class and the remaining classes. The top performing consumer sleep trackers are shown in bold. Abbreviation: *AUROC*, area under receiver operating characteristic curve.
