# Supplementary material for: Accuracy of 11 Wearable, Nearable, and Airable Consumer Sleep Trackers: Prospective Multicenter Validation Study
Source: JMIR Mhealth Uhealth. 2023 Nov 2;11:e50983. doi: 10.2196/50983 (PMC10654909; doi:10.2196/50983)
Supplement: Multimedia Appendix 5 [file mhealth_v11i1e50983_app5.pdf]

Multimedia Appendix 5. Epoch-by-epoch agreement: deep stage classification.

Device	Sensitivity	Specificity	Accuracy	F1 score	Weighted F1	AUROC
**Airable**						
SleepRoutine (67)	0.6712	0.8973	0.8725	0.5355	0.8833	0.7842
SleepScore (38)	0.5247	0.8264	0.7933	0.3574	0.8200	0.6755
Pillow (74)	0.8594	0.4449	0.4898	0.2673	0.5717	0.6522
**Nearable**						
Withings Sleep Tracking Mat (75)	0.5633	0.8270	0.7981	0.3800	0.8245	0.6952
Google Nest Hub 2 (33)	0.1308	0.8883	0.8102	0.1245	0.8142	0.5096
Amazon Halo Rise (28)	0.5467	0.9018	0.8647	0.4575	0.8742	0.7242
**Wearable**						
Google Pixel Watch (30)	0.6937	0.9290	**0.9057**	**0.5922**	**0.9117**	0.8113
Galaxy Watch 5 (22)	0.4752	**0.9481**	0.8982	0.4963	0.8962	0.7117
Fitbit Sense 2 (26)	0.6710	0.9247	0.9013	0.5564	0.9087	**0.7979**
Apple Watch 8 (26)	0.4130	0.8412	0.7937	0.3073	0.8155	0.6271
Oura Ring 3 (53)	**0.7784**	0.7974	0.7955	0.4272	0.8316	0.7879

Deep stage classification was used to classify the deep class and the remaining classes. The top performing consumer sleep trackers are shown in bold. Abbreviation: *AUROC*, area under receiver operating characteristic curve.
