# Supplementary material for: Accuracy of 11 Wearable, Nearable, and Airable Consumer Sleep Trackers: Prospective Multicenter Validation Study
Source: JMIR Mhealth Uhealth. 2023 Nov 2;11:e50983. doi: 10.2196/50983 (PMC10654909; doi:10.2196/50983)
Supplement: Multimedia Appendix 6 [file mhealth_v11i1e50983_app6.pdf]

Multimedia Appendix 6. Epoch-by-epoch agreement: rapid eye movement stage classification.

Device	Sensitivity	Specificity	Accuracy	F1 score	Weighted F1	AUROC
**Airable**						
SleepRoutine (67)	0.7394	**0.9609**	**0.9258**	**0.7596**	**0.9250**	**0.8502**
SleepScore (38)	0.4587	0.7895	0.7409	0.3418	0.7658	0.6241
Pillow (74)	0.1140	0.9126	0.7874	0.1440	0.7635	0.5133
**Nearable**						
Withings Sleep Tracking Mat (75)	0.3964	0.8906	0.8127	0.4001	0.8120	0.6435
Google Nest Hub 2 (33)	0.1805	0.8514	0.7397	0.1876	0.7356	0.5159
Amazon Halo Rise (28)	**0.7490**	0.9401	0.9090	0.7283	0.9100	0.8445
**Wearable**						
Google Pixel Watch (30)	0.6548	0.9029	0.8608	0.6146	0.8641	0.7788
Galaxy Watch 5 (22)	0.6265	0.9058	0.8590	0.5982	0.8615	0.7661
Fitbit Sense 2 (26)	0.6812	0.9297	0.8906	0.6623	0.8918	0.8055
Apple Watch 8 (26)	0.4276	0.9070	0.8340	0.4394	0.8321	0.6673
Oura Ring 3 (53)	0.7118	0.8716	0.8456	0.5993	0.8549	0.7917

REM stage classification was used to classify the REM class and the remaining classes. The top performing consumer sleep trackers are shown in bold. Abbreviations: *AUROC* area under receiver operating characteristic curve; *REM*, rapid-eye movement sleep.
