# Supplementary material for: Accuracy of 11 Wearable, Nearable, and Airable Consumer Sleep Trackers: Prospective Multicenter Validation Study
Source: JMIR Mhealth Uhealth. 2023 Nov 2;11:e50983. doi: 10.2196/50983 (PMC10654909; doi:10.2196/50983)
Supplement: Multimedia Appendix 7 [file mhealth_v11i1e50983_app7.pdf]

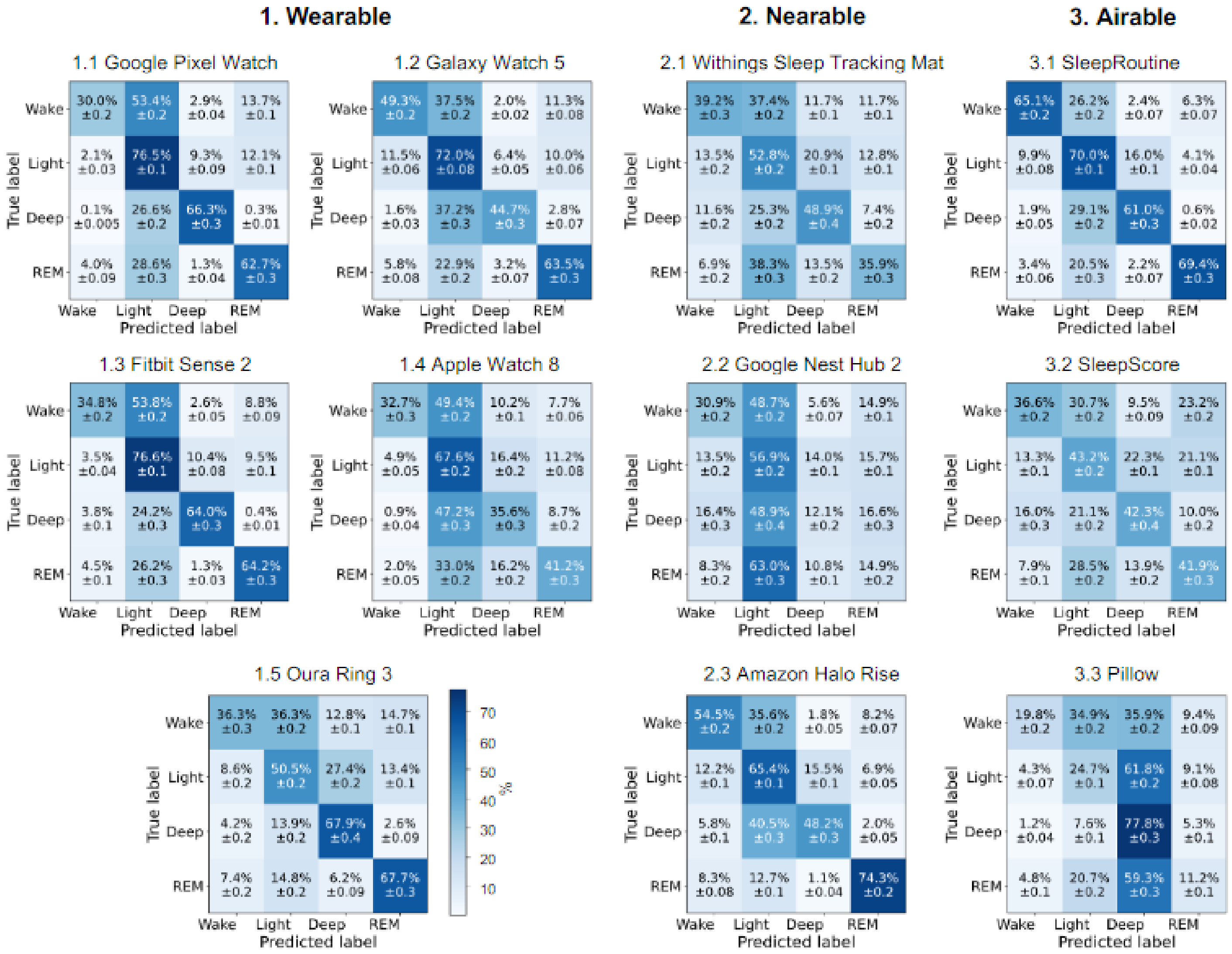

Multimedia Appendix 7. Normalized confusion matrices: mean and variance of predicted values across participants. The mean and variance of 4-stage sleep classification confusion matrices across participants are presented, with comparison of consumer sleep trackers (CSTs). Each row in the confusion matrix is the sleep stage annotated by polysomnography, while each column represents the sleep stage annotated by the CST. REM: rapid eye movement.
