# Supplementary material for: Accuracy of 11 Wearable, Nearable, and Airable Consumer Sleep Trackers: Prospective Multicenter Validation Study
Source: JMIR Mhealth Uhealth. 2023 Nov 2;11:e50983. doi: 10.2196/50983 (PMC10654909; doi:10.2196/50983)
Supplement: Multimedia Appendix 11 [file mhealth_v11i1e50983_app11.pdf]

Multimedia Appendix 11. Epoch-by-epoch agreement: subgroup analysis of the apnea-hypopnea index.

**Device**	**AHI**		**AHI**	
	**≤ 5**	**> 5**	**≤ 30**	**> 30**
**Airable**				
SleepRoutine (67)	0.6317	**0.7073**	**0.6773**	**0.7119**
SleepScore (38)	0.3489	0.4234	0.4099	0.3870
Pillow (74)	0.2731	0.2526	0.2632	0.2398
**Nearable**				
Withings Sleep Tracking Mat (75)	0.4623	0.4434	0.4642	0.3883
Google Nest Hub 2 (33)	0.3505	0.2815	0.3078	0.2564
Amazon Halo Rise (28)	**0.6337**	0.6192	0.6162	0.6567
**Wearable**				
Google Pixel Watch (30)	0.5972	0.5559	0.5529	0.6141
Galaxy Watch 5 (22)	0.5803	0.5741	0.5753	0.5868
Fitbit Sense 2 (26)	0.6216	0.5611	0.5721	0.6179
Apple Watch 8 (26)	0.4303	0.5290	0.5279	0.2170
Oura Ring 3 (53)	0.5506	0.5097	0.5165	0.5261

Values of the top-performing CSTs are shown in bold. Abbreviations: *AHI,* apnea-hypopnea index; *CST,* consumer sleep tracker.
