# Supplementary material for: Accuracy of 11 Wearable, Nearable, and Airable Consumer Sleep Trackers: Prospective Multicenter Validation Study
Source: JMIR Mhealth Uhealth. 2023 Nov 2;11:e50983. doi: 10.2196/50983 (PMC10654909; doi:10.2196/50983)
Supplement: Multimedia Appendix 12 [file mhealth_v11i1e50983_app12.pdf]

Multimedia Appendix 12. Epoch-by-epoch agreement: subgroup analysis of the apnea-hypopnea index and demographic characteristics in Seoul National University Bundang Hospital.

	**Apnea-Hypopnea Index**		**Sleep Efficiency**		**Body Mass Index**		**Gender**	
	**≤ 15**	**> 15**	**≤ 85%**	**> 85%**	**≤ 25**	**> 25**	**Male**	**Female**
**Airable**								
SleepRoutine (29)	**0.7032**	**0.7259**	**0.7004**	**0.7000**	**0.7281**	**0.7036**	**0.7115**	**0.7212**
SleepScore (26)	0.4120	0.4646	0.4350	0.4316	0.4341	0.4476	0.4554	0.4081
Pillow (37)	0.2817	0.2451	0.2398	0.2729	0.2478	0.2751	0.2576	0.2567
**Nearable**								
Withings Sleep Tracking Mat (37)	0.4246	0.4109	0.3605	0.4557	0.4475	0.3791	0.4255	0.3913
Google Nest Hub 2 (14)	0.2281	0.3030	0.2671	0.2386	0.2801	0.2259	0.2555	0.2476
Amazon Halo Rise (12)	0.6250	0.6121	0.6268	0.5743	0.6340	0.5814	0.6031	0.6782
**Wearable**								
Google Pixel Watch (12)	0.5379	0.5347	0.2671	0.2386	0.5125	0.5945	0.5071	0.6099
Galaxy Watch 5 (3)	0.6317	0.6226	0.6516	0.5738	0.6317	0.6226	0.6516	0.5738
Fitbit Sense 2 (9)	0.4868	0.5244	0.4501	0.5491	0.4926	0.5431	0.4941	0.5887
Apple Watch 8 (8)	0.6283	0.4341	0.5001	0.5700	0.6204	0.4677	0.5212	0.5965
Oura Ring 3 (22)	0.5599	0.4886	0.5078	0.5076	0.5386	0.4864	0.4939	0.5661

The number in the parenthesis indicates the number of participants tested with each device. Values for the top-performing consumer sleep trackers are shown in bold. Abbreviations: SNUBH, Seoul National Bundang Hospital; AHI, apnea-hypopnea index.
