# Supplementary material for: Accuracy of 11 Wearable, Nearable, and Airable Consumer Sleep Trackers: Prospective Multicenter Validation Study
Source: JMIR Mhealth Uhealth. 2023 Nov 2;11:e50983. doi: 10.2196/50983 (PMC10654909; doi:10.2196/50983)
Supplement: Multimedia Appendix 13 [file mhealth_v11i1e50983_app13.pdf]

Multimedia Appendix 13. Epoch-by-epoch agreement: subgroup analysis of the apnea-hypopnea index and demographic characteristics in Clionic Lifecare Clinic.

	**Apnea-Hypopnea Index**		**Sleep Efficiency**		**Body Mass Index**		**Gender**	
	**≤ 15**	**> 15**	**≤ 85%**	**> 85%**	**≤ 25**	**> 25**	**Male**	**Female**
**Airable**								
SleepRoutine (38)	**0.6278**	**0.7507**	**0.6769**	**0.6164**	**0.6541**	**0.6582**	**0.7178**	**0.6228**
SleepScore (12)	0.2920	0.3743	0.2401	0.2961	0.3297	0.2843	0.2594	0.3147
Pillow (37)	0.2427	0.2947	0.2635	0.2428	0.2724	0.2155	0.2996	0.2344
**Nearable**								
Withings Sleep Tracking Mat (38)	0.4859	0.4601	0.4176	0.4722	0.4990	0.4356	0.5436	0.4568
Google Nest Hub 2 (19)	0.3325	0.3149	0.3463	0.3006	0.3542	0.2489	0.4100	0.3142
Amazon Halo Rise (16)	0.5868	0.6473	0.6463	0.5905	0.6164	0.5515	0.5924	0.6113
**Wearable**								
Google Pixel Watch (18)	0.5819	0.6402	0.5499	0.6280	0.6109	0.5716	0.6045	0.5889
Galaxy Watch 5 (19)	0.5617	0.5543	0.5757	0.5523	0.5444	0.6232	0.5396	0.5900
Fitbit Sense 2 (17)	0.6118	0.6886	0.5767	0.6521	0.6438	0.5641	0.6773	0.6182
Apple Watch 8 (18)	0.3765	0.5811	0.1568	0.4626	0.4263	0.4020	0.4990	0.3466
Oura Ring 3 (31)	0.5176	0.5244	0.4636	0.5360	0.5381	0.4789	0.4936	0.5316

The number in the parenthesis indicates the number of participants tested with each device. Values for the top-performing consumer sleep trackers are shown in bold. Abbreviations: CLC, Clionic Life Center; AHI, apnea-hypopnea index.
