# Supplementary material for: Accuracy of 11 Wearable, Nearable, and Airable Consumer Sleep Trackers: Prospective Multicenter Validation Study
Source: JMIR Mhealth Uhealth. 2023 Nov 2;11:e50983. doi: 10.2196/50983 (PMC10654909; doi:10.2196/50983)
Supplement: Multimedia Appendix 14 [file mhealth_v11i1e50983_app14.pdf]

Multimedia Appendix 14. Group-averaged macro F1 scores: subgroup analysis of the apnea-hypopnea index and demographic characteristics in Seoul National University Bundang Hospital.

	**AHI**			**Sleep Efficiency**			**Body Mass Index**			**Gender**		
	**≤ 15**	**> 15**	**P**	**≤ 85%**	**> 85%**	**P**	**≤ 25**	**> 25**	**P**	**Male**	**Female**	**P**
**Airable**												
SleepRoutine (29)	0.67±0.04(10)	0.67±0.15(19)	0.9381	0.66±0.09(16)	0.69±0.15(13)	0.6385	0.68±0.09(14)	0.66±0.15(15)	0.6846	0.66±0.14(22)	0.71±0.05(7)	0.4452
SleepScore (26)	0.38±0.11(11)	0.44±0.19(15)	0.3637	0.41±0.19(14)	0.42±0.11(12)	0.9381	0.4±0.19(16)	0.44±0.1(10)	0.6355	0.43±0.17(20)	0.36±0.1(6)	0.3281
Pillow (37)	0.24±0.09(15)	0.22±0.1(22)	0.5421	0.21±0.1(20)	0.25±0.08(17)	0.2305	0.22±0.09(21)	0.25±0.09(16)	0.3980	0.24±0.1(27)	0.21±0.09(10)	0.5478
**Nearable**												
Withings Sleep Tracking Mat (37)	0.38±0.16(15)	0.37±0.18(22)	0.8213	0.32±0.14(20)	0.44±0.18(17)	**0.0234**	0.4±0.17(21)	0.34±0.17(16)	0.3632	0.38±0.16(27)	0.36±0.2(10)	0.7777
Google Nest Hub 2 (14)	0.22±0.08(6)	0.23±0.07(8)	0.7547	0.24±0.1(7)	0.21±0.05(7)	0.5345	0.23±0.09(9)	0.21±0.04(5)	0.6945	0.22±0.09(10)	0.22±0.05(4)	0.9790
Amazon Halo Rise (12)	0.56±0.05(5)	0.56±0.17(7)	0.9850	0.56±0.06(7)	0.56±0.2(5)	0.9373	0.57±0.06(6)	0.54±0.18(6)	0.7295	0.55±0.15(9)	0.6±0.07(3)	0.6182
**Wearable**												
Google Pixel Watch (12)	0.52±0.11(5)	0.51±0.15(7)	0.9389	0.47±0.18(5)	0.55±0.07(7)	0.3246	0.49±0.16(8)	0.57±0.05(4)	0.3853	0.5±0.15(9)	0.57±0.06(3)	0.4443
Galaxy Watch 5 (3)	0.63±0.0(1)	0.62±0.05(2)	-	0.65±0.02(2)	0.57±0.0(1)	-	0.63±0.0(1)	0.62±0.05(2)	-	0.65±0.02(2)	0.57±0.0(1)	-
Fitbit Sense 2 (9)	0.48±0.01(3)	0.52±0.13(6)	0.7147	0.45±0.15(3)	0.53±0.05(6)	0.2957	0.48±0.13(5)	0.54±0.05(4)	0.5084	0.49±0.11(7)	0.57±0.04(2)	0.3545
Apple Watch 8 (8)	0.53±0.14(4)	0.43±0.23(4)	0.5212	0.46±0.22(6)	0.55±0.09(2)	0.6473	0.51±0.1(4)	0.46±0.25(4)	0.7544	0.51±0.22(6)	0.41±0.05(2)	0.6228
Oura Ring 3 (22)	0.54±0.1(7)	0.41±0.14(15)	0.0613	0.43±0.13(11)	0.48±0.14(11)	0.4390	0.45±0.16(12)	0.45±0.12(10)	0.9283	0.42±0.14(16)	0.53±0.1(6)	0.1112

The number in the parenthesis indicates the number of participants tested with each device. Mean ± standard deviation of macro-averaged F1 scores across participants. The bold values represent statistical significance ($p < 0.05$). Abbreviations: *SNUBH*, Seoul National Bundang Hospital; *AHI*, Apnea-Hypopnea Index.
