# Supplementary material for: Accuracy of 11 Wearable, Nearable, and Airable Consumer Sleep Trackers: Prospective Multicenter Validation Study
Source: JMIR Mhealth Uhealth. 2023 Nov 2;11:e50983. doi: 10.2196/50983 (PMC10654909; doi:10.2196/50983)
Supplement: Multimedia Appendix 15 [file mhealth_v11i1e50983_app15.pdf]

Multimedia Appendix 15. Group-averaged macro F1 scores: subgroup analysis of the apnea-hypopnea index and demographic characteristics in Clionic Lifecare Clinic.

	**AHI**			**Sleep Efficiency**			**Body Mass Index**			**Gender**		
	**≤ 15**	**> 15**	**P**	**≤ 85%**	**> 85%**	**P**	**≤ 25**	**> 25**	**P**	**Male**	**Female**	**P**
**Airable**												
SleepRoutine (38)	0.54±0.14(30)	0.68±0.07(8)	**0.012**	0.57±0.19(9)	0.57±0.12(29)	0.9499	0.57±0.13(28)	0.59±0.16(10)	0.6541	0.63±0.1(12)	0.54±0.14(26)	0.0587
SleepScore (12)	0.25±0.11(10)	0.37±0.13(2)	0.2657	0.21±0.02(3)	0.29±0.14(9)	0.4221	0.31±0.16(6)	0.23±0.04(6)	0.2963	0.24±0.0(2)	0.28±0.14(10)	0.7527
Pillow (37)	0.22±0.1(30)	0.22±0.06(7)	0.9687	0.23±0.13(9)	0.22±0.08(28)	0.9326	0.23±0.09(27)	0.2±0.11(10)	0.3346	0.23±0.07(12)	0.22±0.11(25)	0.6755
**Nearable**												
Withings Sleep Tracking Mat (38)	0.42±0.15(30)	0.38±0.14(8)	0.5694	0.34±0.16(9)	0.43±0.14(29)	0.0952	0.42±0.15(28)	0.38±0.16(10)	0.5591	0.46±0.11(12)	0.39±0.16(26)	0.1826
Google Nest Hub 2 (19)	0.3±0.11(16)	0.28±0.03(3)	0.7758	0.31±0.13(6)	0.29±0.09(13)	0.7409	0.32±0.08(14)	0.24±0.13(5)	0.1788	0.38±0.06(2)	0.29±0.11(17)	0.2574
Amazon Halo Rise (16)	0.55±0.07(13)	0.63±0.02(3)	0.0835	0.65±0.0(1)	0.56±0.07(15)	-	0.57±0.07(12)	0.54±0.07(4)	0.5254	0.56±0.08(8)	0.57±0.06(8)	0.6740
**Wearable**												
Google Pixel Watch (18)	0.55±0.18(15)	0.59±0.09(3)	0.7245	0.47±0.25(6)	0.59±0.07(12)	0.1535	0.57±0.08(13)	0.5±0.29(5)	0.4219	0.6±0.11(2)	0.55±0.17(16)	0.6642
Galaxy Watch 5 (19)	0.51±0.11(14)	0.52±0.09(5)	0.8354	0.44±0.11(3)	0.52±0.1(16)	0.2216	0.48±0.11(14)	0.6±0.05(5)	**0.0304**	0.49±0.1(10)	0.53±0.11(9)	0.4871
Fitbit Sense 2 (17)	0.56±0.17(14)	0.63±0.09(3)	0.5236	0.51±0.24(6)	0.61±0.07(11)	0.2346	0.59±0.07(13)	0.49±0.29(4)	0.3002	0.66±0.06(2)	0.56±0.17(15)	0.4262
Apple Watch 8 (18)	0.38±0.19(14)	0.57±0.2(4)	0.1072	0.15±0.06(2)	0.45±0.19(16)	**0.0490**	0.43±0.21(13)	0.4±0.21(5)	0.8432	0.49±0.21(9)	0.35±0.17(9)	0.1471
Oura Ring 3 (31)	0.48±0.13(25)	0.52±0.11(6)	0.4699	0.44±0.2(9)	0.5±0.06(22)	0.1852	0.49±0.09(23)	0.48±0.19(8)	0.9234	0.47±0.09(9)	0.49±0.13(22)	0.6314

The number in the parenthesis indicates the number of participants tested with each device. Mean ± standard deviation of macro-averaged F1 scores across participants. The bold values represent statistical significance ($p < 0.05$). Abbreviations: *CLC,* Clionic Life Center; *AHI,* Apnea-Hypopnea Index.
