# Supplementary material for: Accuracy of 11 Wearable, Nearable, and Airable Consumer Sleep Trackers: Prospective Multicenter Validation Study
Source: JMIR Mhealth Uhealth. 2023 Nov 2;11:e50983. doi: 10.2196/50983 (PMC10654909; doi:10.2196/50983)
Supplement: Multimedia Appendix 16 [file mhealth_v11i1e50983_app16.pdf]

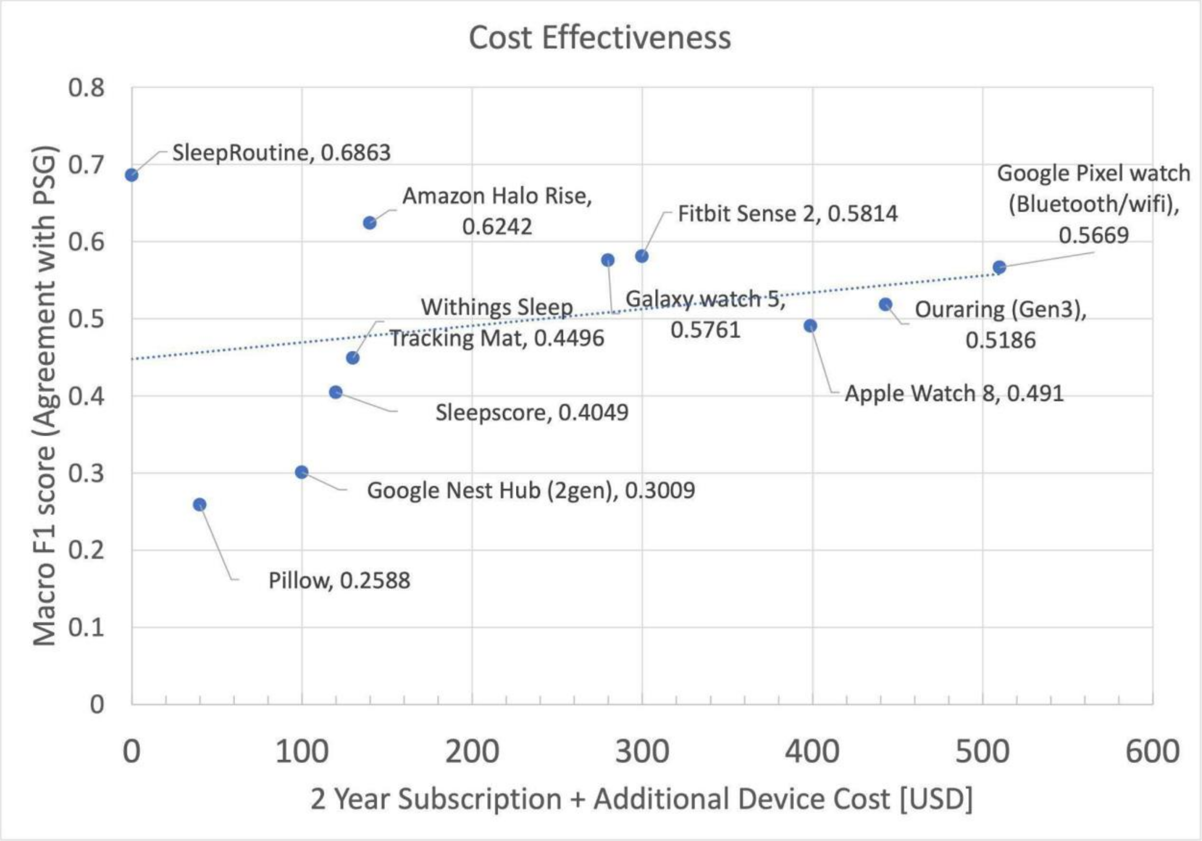


**Multimedia Appendix 16.** Cost-effectiveness of consumer sleep trackers. Relationship between the sum of the 2-year subscription fees and the cost of purchasing additional devices is shown, compared with the corresponding F1 scores. By plotting the total cost against the F1 score, we determined the cost-performance profiles of different sleep trackers.

Pricing references

- Pillow: https://apps.apple.com/gy/app/pillow-auto-sleep-tracker/id878691772
- Withings Sleep Traking Mat: https://www.withings.com/us/en/sleep/shop
- Sleepscore: https://apps.apple.com/us/app/sleepscore/id1364781299
- SleepRoutine: https://apps.apple.com/kr/app/sleep-routine-tracker-alarm/id1620995257?l=en
- Google Nest Hub: https://store.google.com/us/product/nest_hub_2nd_gen?hl=en-US&pli=1
- Amazon Halo Rise (discontinued): https://press.aboutamazon.com/2022/9/amazon-introduces-halo-rise-a-first-of-its-kind-bedside-sleep-tracker
- Google Pixel Watch: https://store.google.com/us/product/google_pixel_watch?hl=en-US
- Galaxy Watch 5: https://www.samsung.com/us/watches/galaxy-watch5/buy/
- Fitbit Sense 2: https://www.fitbit.com/global/us/products/smartwatches/sense2
- Apple Watch 8: https://www.apple.com/apple-watch-series-8/why-apple-watch/
- Ouraring (Gen3): https://ouraring.com/product/heritage-silver
